# The role of eye movements and covert shifts of attention in working and long-term memory retrieval

**DOI:** 10.3758/s13423-026-02945-0

**Published:** 2026-06-15

**Authors:** Ruhi Bhanap, Klaus Oberauer, Agnes Rosner

**Affiliations:** 1 Department of Psychology, University of Zurich, Zurich, Switzerland; 2 Faculty of Psychology, Dresden University of Technology, Dresden, Germany; 3 Department of Psychology, Leibniz University Hannover, Hanover, Germany; 4 Faculty of Psychology, Technische Universität Dresden, Chemnitzer Strasse 46b, 01187 Dresden, Germany

**Keywords:** Working memory, Eye movements, Covert attention

## Abstract

The “Looking at Nothing” (LAN) effect describes the tendency to look toward locations where information was originally presented during memory retrieval. Previous research by Scholz et al. (*Memory & Cognition*, 46, 230-243, 2018) suggested that memory performance may benefit not only from eye movements to these locations, but also from covert shifts of attention. The present study investigated whether such effects also occur during retrieval from working memory. Across two experiments, participants encoded three word pairs presented at distinct screen locations. Memory for one word pair was later tested using auditory cues, either immediately after encoding (Experiments 1 and 2) or after a delay (Experiment 2). During retrieval, either gaze location (Experiments 1 and 2) or covert attention (Experiment 1) was manipulated using a digit-tracking task presented at one of three locations: congruent with the original word-pair location, incongruent, or central.Results showed no differences in memory performance between conditions. Thus, directing gaze or covert attention toward the original presentation location did not facilitate retrieval. These findings suggest that spatial reinstatement may not enhance working- or long-term memory performance in associative recognition tasks involving word pairs.

## Introduction

The Looking at Nothing (LAN) effect refers to the tendency to look towards the empty locations where information was originally presented, during later retrieval (Scholz et al., 2016; Spivey & Geng, 2001; Wynn et al., 2022). In typical paradigms, participants encode items shown in specific screen positions and later retrieve them while those positions remain visible only as an empty grid. Despite the absence of visual input, gaze is spontaneously directed back to the associated locations. Theoretical accounts differ in whether these eye movements merely reflect retrieval or play a functional role.

The external memory account (Richardson & Spivey, 2000) proposes that only partial visual information is encoded together with spatial pointers. When additional information is needed, people look back to the original location to obtain more information. During LAN, however, the location is empty, so these eye movements provide no new input and are considered epiphenomenal.

In contrast, Ferreira et al. (2008) proposed a functional account in which visual, spatial, and verbal aspects of an event are tightly integrated. Activating one component at retrieval triggers retrieval of others in a feedback–feedforward cycle, allowing location to serve as a retrieval cue. Eye movements are therefore part of a mechanism that can enhance memory performance. A related proposal stems from scanpath theory (Noton & Stark, 1971). According to Wynn et al. (2021) and Johansson et al. (2022), the sequence of eye movements generated during encoding is stored with the memory representation; reinstating this scanpath during retrieval produces LAN and facilitates access to stored information.

Empirical evidence for a functional benefit is mixed. Several studies report positive relations between LAN magnitude and memory accuracy (Laeng & Teodorescu, 2002; Olsen et al., 2016; Wynn et al., 2022), whereas others find no association (Kumcu & Thompson, 2020; Martarelli & Mast, 2013). Only a few experiments have manipulated gaze direction to test causality. Free viewing during retrieval often leads to better memory than enforced central fixation, supporting a functional interpretation (Johansson et al., 2012; Laeng et al., 2014), although this advantage may partly reflect increased cognitive load under fixation.

More direct tests have compared performance when gaze is directed to congruent versus incongruent locations. Johansson and Johansson (2014) observed higher accuracy and faster responses for congruent locations in spatial-relation judgments, and Scholz et al. (2016) replicated this effect with verbal materials, though alternative results exist (Wantz et al., 2016). Scholz et al. (2018) further examined whether covert attention, rather than overt eye movements, drives the effect. Using a digit-tracking task to manipulate overt or covert attention, they found memory benefits for congruent locations in both conditions, suggesting that directing attention to the original location supports retrieval.

LAN research has primarily focused on episodic memory, using study materials that far exceeded the capacity of working memory (WM). Recent evidence from Bhanap et al. (2025a) demonstrates that LAN also occurs during WM retrieval. Therefore, in the present study we carried out two experiments to address the question: Is LAN functional in WM as well?

In Experiment 1, we carried out a conceptual replication of Scholz et al. (2018), manipulating overt and covert shifts of attention in a WM task in which we had previously observed LAN. In Experiment 2, we investigated if presence of functionality of LAN depends on whether the retrieval taps into WM or long-term memory (LTM). Our primary aim was to examine whether the mere presence of LAN necessarily implies that it serves a functional role. Although many studies have documented LAN, far fewer have tested its functional significance, and only a small subset has demonstrated a causal functional effect.

A key similarity between Scholz et al. and Bhanap et al. is that both studies used associative recognition tasks with verbal materials, but they differ in the memory systems they predominantly tap into. Scholz et al. (2018) required participants to remember four sentences, each with four attributes, a task that relies on episodic long-term memory (eLTM). In contrast, Bhanap et al. (2025a, 2025b) used sets of three word pairs. Short-term retention of up to three pairs is handled predominantly by WM, as shown by the absence of proactive interference at this set size (Bartsch & Oberauer, 2023).

WM maintains task-relevant information in a highly accessible state, whereas retrieval from eLTM is slower (Oberauer, 2002). Many accounts of LAN functionality assume a sequence of feedforward and feedback processes: retrieving an item triggers a look toward its associated location (the feedforward component), which feeds back to facilitate further retrieval (the feedback component). We assume that whether this feedback loop fully unfolds before a response is given will determine if LAN is functional or not. In eLTM retrieval, the loop likely has time to complete; in WM retrieval, which is faster and less demanding, it may not. Thus, the present study asks whether a situation involving reduced retrieval competition and shorter retrieval times – as is typical in WM – still permits LAN to exert a functional effect.^1^

## Methods

### Participants

We collected data for 28 participants (mean age = 22.60, *SD* = 2.8 years; 22 women, six men). All participants took part in the study at the University of Zurich and received either monetary compensation or course credit. We collected data till the Bayes factor for the main effect of Cue was over 3 in favor of either the null or the alternative hypothesis with respect to the main effect of cue (Schönbrodt et al., 2017). The analysis we used for this cut-off criterion compared models that differed in both the fixed and the random effect of cue. For the analysis reported below we used a more conservative approach that tests fixed effects while holding the random effects constant between models. The participants were native German speakers and had normal or corrected-to-normal vision. They signed an informed consent form, and the experimental protocol was in accordance with the guidelines of the Institutional Review Board of the Faculty of Arts and Social Sciences at the University of Zurich. We planned to exclude participants if their accuracy was below 50%, but none of the participants showed such poor performance.

### Apparatus

Participants were seated 70 cm from the screen with a resolution of 1,920 × 1,080 pixels (50 × 30 cm). Stimuli were presented with MATLAB 2014b with the Psychophysics Toolbox 3 extension. An SMI iView Red 500 Eye Tracker sampled the eye-tracking data from the right eye at a rate of 500 Hz. Eye movements were recorded after a 9-point calibration. During the experiment, participants placed their head on a chin rest.

### Materials

**Fig. 1:**
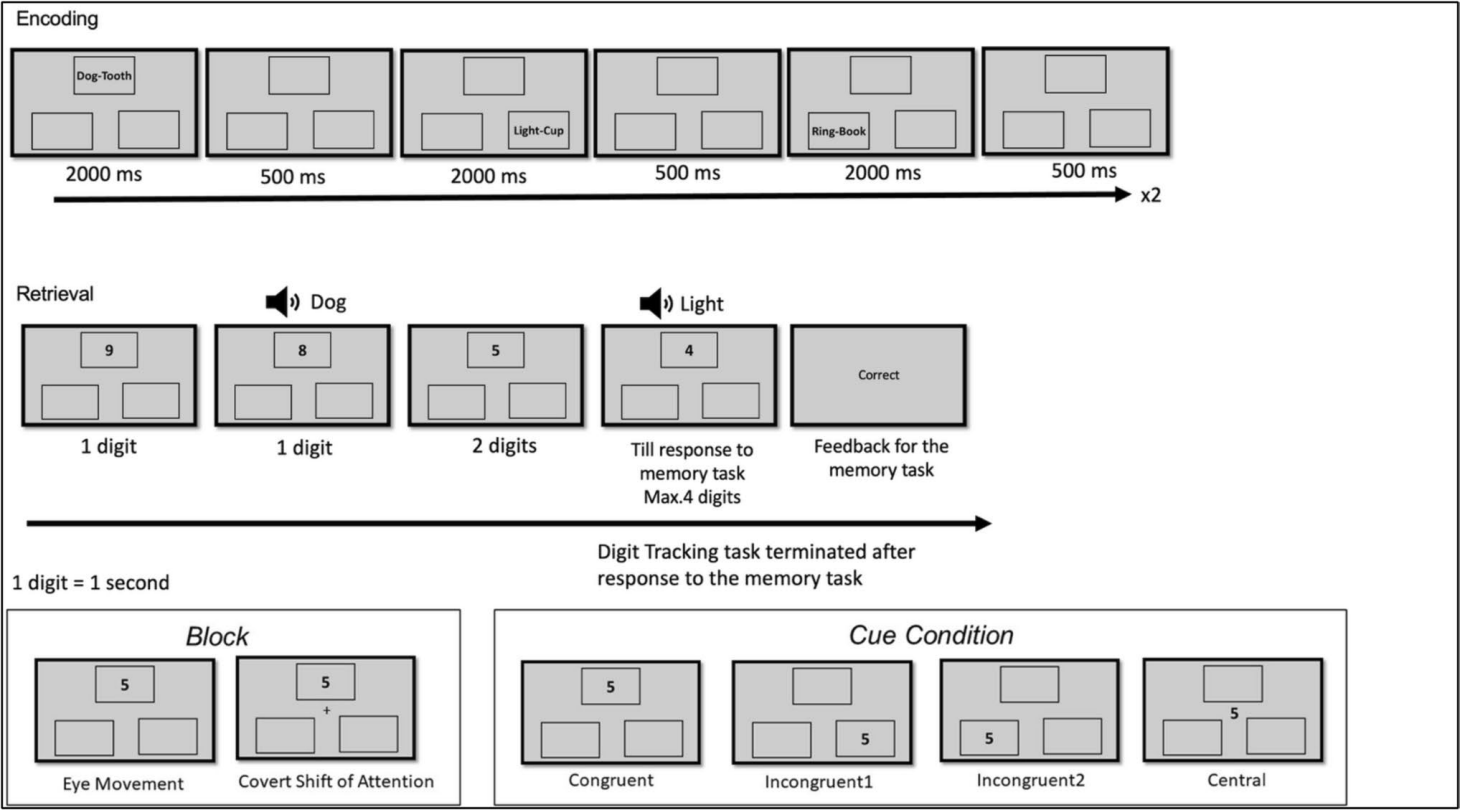
Structure of a trial in the experiment

Participants were presented with three rectangles in a triangular arrangement (see Fig. 1). The size of rectangles was 300 × 200 pixels. The distance from the center of each rectangle to the center of the screen was 10 degrees horizontally and 6 degrees vertically for the bottom rectangles; for the top rectangle, it was 6 degrees vertically. During the encoding phase, participants were presented with three word pairs, each appearing in the center of one of the rectangles. The words were chosen from 768 German words from the dlexdb.de lexical database. The average lemma frequency was 24.76 per million. On each trial, six new words were randomly drawn and paired to form the three words pairs. The font size of the words was 15. During the retrieval phase, participants heard two word probes through the speakers. Simultaneously, they were presented with a series of nine digits and were asked to track the changes in the digits (see Scholz et al., 2018).^2^

### Procedure

The experiment commenced with two practice trials, followed by 96 experimental trials, each following an identical procedure comprising an encoding and a retrieval phase, as illustrated in Fig. 1. The experiment was structured into two blocks. In the covert shift of attention (CA) block, participants were instructed to maintain their gaze at the center of the screen throughout the retrieval phase. Conversely, in the eye movement (EM) block, participants were allowed to move their eyes freely. Each trial began with a fixation cross displayed at the center of the screen for 1,000 ms. During the encoding phase, participants were shown three pairs of words, with each pair appearing in one of three rectangles on the screen. The word pairs were always presented sequentially in a clockwise direction, starting from the top rectangle and ending at the bottom left. This consistent order of presentation could establish a top-down attentional set, enhancing the encoding process (Prinzmetal et al., 2010). Each word pair was displayed for 2,000 ms, with a 500-ms interval between pairs. The encoding phase was repeated twice to ensure that participants thoroughly encoded the word pairs and their respective locations (Johansson & Johansson  2014). Before the retrieval phase, a fixation cross was again shown at the center of the screen for 1,000 ms. Throughout the trial, the three empty rectangles remained visible on screen.

During the retrieval phase, participants heard two words with a 2,000-ms gap between them. These words could either belong to the same word pair within the same rectangle (positive trials) or to different word pairs in different rectangles (lure trials). After hearing the second word, participants indicated their response via a keypress: the left arrow key for words from the same pair, and the right arrow key for words from different pairs. They had up to 4 s to respond, though the trial would end as soon as a key was pressed. After each trial, participants received feedback on the accuracy of their response to the memory task.

At the start of the retrieval phase, a sequence of nine digits was presented, with each digit shown for 1 s before the next appeared. Participants were instructed to press the space bar to indicate a change in the digit, which led to the digit changing from white to a shade of gray to indicate that they had detected the change successfully. If the space bar was not pressed, the digit remained white and changed to the next one after 1 s. In each trial, the digit stream appeared in one of four possible locations: one of the three rectangles or the center of the screen. The location of the digit stream could be either congruent or incongruent with the location of the word pair associated with the first word probe, or it could be centered. The incongruent locations were termed Incongruent1 and Incongruent2, depending on whether they were in the clockwise or counterclockwise direction relative to the congruent location. Participants were instructed to press the space bar whenever the digit changed. If they failed to follow the block-specific instructions – for example, not focusing on the center in the CA block or fixating on the center in the EM block – the trial would pause, and the screen would flash red for 500 ms as a reminder to adhere to the instructions. After every 16 trials, a re-calibration procedure was conducted. Eye-movement recording began after the practice trials and continued until the end of the experiment.

### Data analysis

We analyzed fixation proportions, response accuracies, and reaction times (RTs). The data analysis was conducted in R with RStudio (R Core Team, 2024). For the eye-movement analysis, the events, fixations, saccades, and blinks were detected through the event detector software provided by SMI. For further analysis, we only used fixations from the right eye. The screen was divided into four areas of interest (AOIs). Each AOI was drawn around the three rectangles and the center of the screen. The size of the AOIs drawn around the three rectangles was 860 × 440 pixels. For the center of the screen, to avoid overlap with other AOIs, we drew an AOI that extended 100 pixels horizontally and vertically from the screen center in both directions.

For the eye-movement block, the AOI in which the digit task appeared was considered the relevant location for that trial. For the covert shift of attention block, the AOI for the center was always coded as the relevant location. Fixation proportions for each trial and participant were calculated as the sum of fixation durations at the relevant AOI divided by the total duration of looking at all four AOIs. As we had four AOIs, 25% indicates the chance level fixation proportion to an area. Therefore, any trials for which the fixation proportion to the relevant location was less than 25% were excluded from the analysis. After that exclusion we included 83.4% of trials for the EM Block and 84.8% for the CA Block. For accuracies and RTs, we ran Bayesian generalized linear models in the BRMS package (Bürkner, 2017). Additionally, for fixation proportions, we ran Bayesian ANOVA in the Bayes Factor package in R with R studio (Morey et al., 2015).

## Results

### Manipulation checks: Eye movements and digit-tracking accuracy

**Fig. 2:**
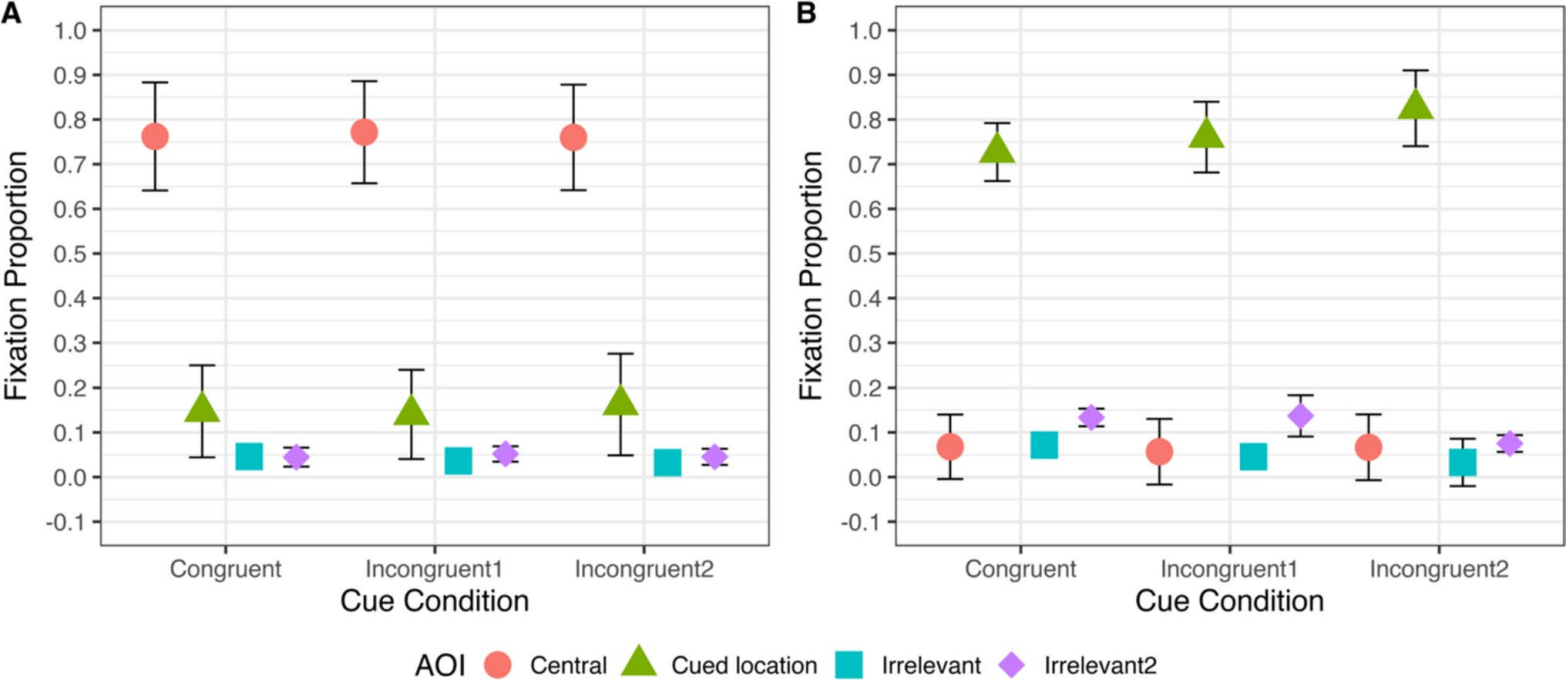
Fixation proportion to the four areas of interest (AOIs) during the retrieval phase. (**A**) covert shift of attention block, (**B**) eye-movement block. The error bars indicate 95% within-subject confidence intervals

We verified to what extent participants followed the instructions, which meant looking at the central location for the CA block and moving their eyes freely and not fixating the central location for the EM block (Fig. 2). We ran a Bayesian ANOVA with fixation proportion as the dependent variable and the location as the fixed effect, with random effects for subject. The location variable was contrast coded (3: central location, −1: the three peripheral AOIs). We compared the model including the fixed effect of location with a model without it while retaining the random effects. Participants looked credibly longer (BF_10_ = 7.5 × 10^88^) at the central location in the CA block. However, descriptively we also observed higher fixation proportions to the cued location than the remaining two locations for the CA block. Thus, there were occasional lapses in maintaining one’s gaze at the central location, and in these cases, the eyes tended to move to the cued location. Participants looked credibly less at the central location as compared to the other AOIs in the EM block (BF_10_ = 6.1 × 10^6^). We additionally looked at whether they looked more at the cued location as compared to the other AOIs in the EM block. We ran a similar analysis with different contrast coding (3: cued location, −1: all the other locations). We found evidence in favor of a main effect of location (BF_10_ = 3.02 × 10^125^), indicating that participants looked more at the cued location as compared to other AOIs.

The mean accuracy for the digit-tracking task for the two blocks was satisfactory: 78.8% (SD = 0.15) for the eye-movement block and 78.1% (SD = 0.15) for the covert shift of attention block. Therefore, the performance in the digit-tracking task was similar in the two blocks, indicating that the conditions were comparably difficult.

#### Memory performance

**Fig. 3:**
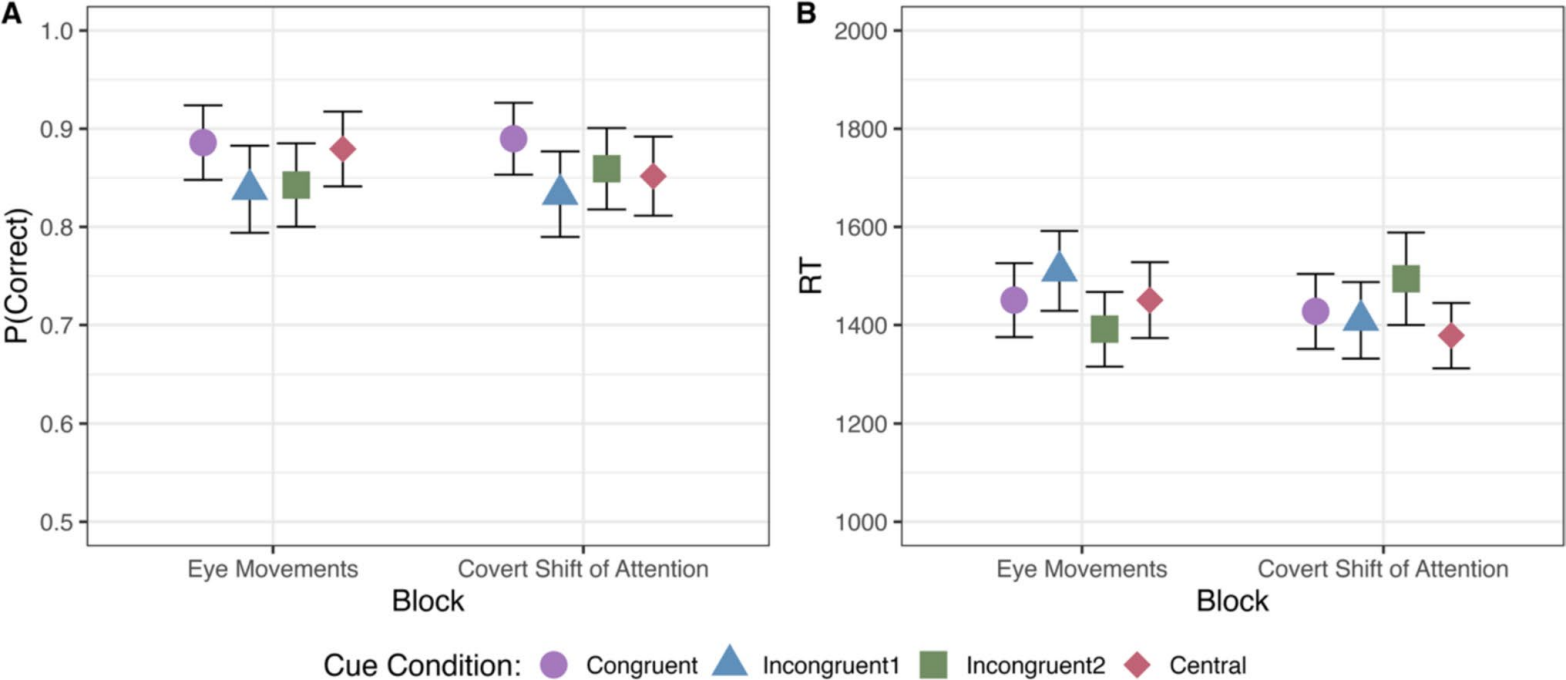
Proportion correct and reaction times (RTs) for the memory task across the cue conditions and blocks. (**A**) Proportion correct and (**B**) RTs. The error bars indicate the 95% within-subject confidence intervals

Figure 3 presents the proportion of correct responses across the two blocks for the four different cue types. To analyze the data, we applied hierarchical Bayesian generalized linear mixed models. Since accuracy for each trial was 0 or 1, we used a binomial model with a logit link function. The regression coefficients were assigned Cauchy priors with a mean of 0 and a scale of 0.353, as recommended by Oberauer (2019). For the random effects, we used Gamma priors with a mean of 1 and a standard deviation of 0.04. The cue variable was contrast coded as follows: 2 for congruent, −1 for incongruent1, −1 for incongruent2, and 0 for central. This coding allowed us to examine whether accuracy in the congruent condition differed from that in the incongruent condition, testing if shifting attention or making eye movements toward the congruent location provided a credible benefit over shifting toward an incongruent location.

To compute the Bayes factor (BF) for each effect, we compared a model including the fixed effect with one excluding it, keeping random effects constant between models. For example, the BF for the main effect of block was calculated by comparing models with and without that fixed effect. Our results showed evidence against a main effect of block (BF_10_ = 0.31), inconclusive evidence for a main effect of cue (BF_10_ = 1.41), and against an interaction between block and cue (BF_10_ = 0.32). We additionally computed BFs for pairwise comparisons between the cue conditions across the two blocks. We pooled the two Incongruent conditions for these comparisons. Table 1 shows that we found inconclusive evidence for a difference for most pairwise comparisons except for the comparison of central and incongruent conditions in the CA block and between central and congruent in the EM block, for which we observed evidence against a difference.

**Table 1:** Bayes factor (BF_10_) for pairwise comparisons of the three cue conditions for the proportion correct for each block

Block	Eye movements		Covert shift of attention	
	Incongruent	Central	Incongruent	Central
Congruent	1.32	0.31	1.32	0.55
Incongruent		0.54		0.29

To see if the benefit of the congruent condition and the cost of the incongruent condition was reflected in the time taken to respond to the task, we analyzed the RTs. We used a shifted lognormal model. The regression coefficients were assigned Gaussian priors with a mean of 0 and a standard deviation of 0.5, the intercept prior with a mean of 7.2 and a standard deviation of 3.5. The priors are set on a log scale. Similar to the accuracy analysis, the cue variable was contrast coded as follows: 2 for congruent, −1 for incongruent1, −1 for incongruent2, and 0 for central. Our results showed evidence against a main effect of block (BF_10_ = 0.17), against a main effect of cue (BF_10_ = 0.02), and against an interaction between block and cue (BF_10_ = 0.01). The BFs for pairwise comparisons are shown in Table 2.

**Table 2:** Bayes factor (BF_10_) for pairwise comparisons for the reaction times across both the cue conditions and blocks

Block	Eye movements		Covert shift of attention	
	Incongruent	Central	Incongruent	Central
Congruent	0.28	0.04	0.05	0.12
Incongruent		0.19		0.08

We found evidence against a difference across all the pairwise comparison. Thus, we did not observe a difference in memory performance between the different cue conditions.

## Discussion

Our study adopted the experimental design of Scholz et al. (2018), who reported enhanced memory performance when eye movements or covert attention were directed to locations congruent with those at encoding, as well as decreased memory performance when they were directed to incongruent locations. In contrast, our results revealed only a weak tendency toward such enhancement, with the evidence remaining ambiguous when analyzing accuracies and conclusive evidence against an effect when analyzing RTs. Moreover, we found evidence against a performance decrease from incongruent cues in the covert shift of attention block, and the evidence for a decrease in the eye-movement block was likewise ambiguous.

**Fig. 4:**
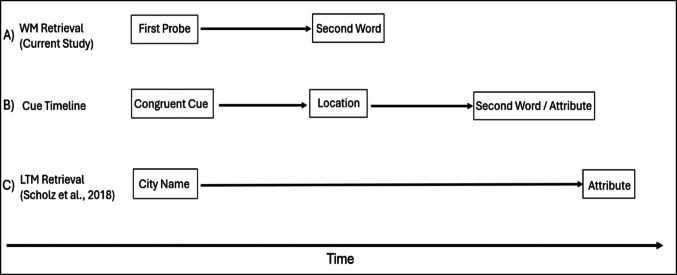
Possible explanation for the results of the experiment. Path (**A**) represents the timeline of the retrieval process in the current study, after the participants hear the first probe, which leads to the retrieval of the second word associated with it through a pairwise binding. Path (**B**) represents the timeline for the indirect path of the congruent cue leading to the activation of location at which it is presented leading to the activation of the second word (current study) and the attribute (Scholz et al., 2018). Path (**C**) represents the timeline of the retrieval process in Scholz et al. (2018) when they hear the city name, and it leads to the activation of the attributes associated to it

We propose that LAN functionality depends on the relative speed of two retrieval pathways: a direct and an indirect location-based path (Fig. 4). In LAN paradigms, spatial location is not task relevant, and retrieval is cued by non-spatial features. Retrieval can therefore occur either directly via cue–target associations or indirectly via activation of the associated location. The indirect path can facilitate performance only if its activation arrives before the direct path has already produced a response. In episodic memory tasks, direct retrieval is often relatively slow due to competition among multiple memory traces, allowing location-based activation to enhance retrieval. In contrast, in our WM task, each cue was associated with a single target, enabling rapid direct retrieval that likely completed before location-based activation could strongly influence performance. To examine whether our findings replicate and to test for a potential beneficial effect of LAN on long-term memory, as proposed, we conducted Experiment 2.

## Experiment 2

In Experiment 2, we tested whether we could observe LAN in an LTM retrieval task with word pairs to explore the timeline-based explanation for the results of Experiment 1. To this end, we made some changes to the experiment. First, every trial consisted of a WM phase, a distractor task, and an LTM phase. In this way we aimed to replicate the results of Experiment 1 and to test the prediction that retrieval from LTM takes longer than retrieval from WM and thus benefits from attention to the congruent location. As Experiment 2 aimed to compare LAN functionality between WM and LTM retrieval tasks, we retained only the eye-movement condition and excluded the covert attention-shift condition.

### Methods

#### Participants

We collected data for 27 participants (mean age = 22.03, *SD* = 1.95 years; 23 women, four men). The exclusion criteria and were the same as in Experiment 1. We planned to exclude participants if their accuracy was below 50%, and five participants had accuracy less than that, so our final sample size was 22 participants.

#### Materials

**Fig. 5:**
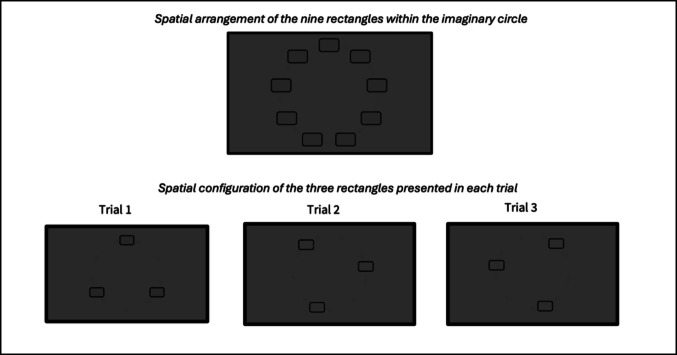
Configuration of the rectangles in Experiment 2

The word materials and the procedure for forming word pairs were identical to those used in Experiment 1. The main modification concerned the locations of the word pairs. The trials with WM tests were organized into mini-blocks of three trials, followed by an LTM test. In each trial of a mini-block, participants were presented with three word pairs, each shown in a different spatial location. Across the three trials, nine unique locations were used so that each word pair tested in the LTM phase was also associated with a unique location. This was done to give the assumed indirect retrieval path – from the cue word to the location to the target word – a chance to be effective in the LTM test by minimizing competition with other word pairs. The nine locations were positioned equidistantly on an imaginary circle with a radius of 11 cm (Fig. 5). To select three non-overlapping locations per trial, for each subset we used three locations forming an equilateral trial, that is, we used locations three steps apart from each other. For instance, the first trial used locations 1, 4, and 7; the second trial used locations 2, 5, and 8, and the third trial used locations 3, 6, and 9. This procedure ensured that each trial presented a new set of three rectangles arranged in a triangular pattern comparable to that used in Experiment 1. During any given trial, participants saw only the three rectangles in which the word pairs appeared.

After three trials with WM tests, participants completed a 45-s distractor task in which they judged whether simple multiplicative equations were correct (e.g., “2 × 5 = 15?”). This served to erase WM representations for the word pairs before the LTM test. As in Experiment 1, retrieval involved a digit-tracking task presented in one of the locations, which could be congruent, incongruent, or presented in the centre.

#### Procedure

The experiment began with three practice trials, followed by 24 experimental mini-blocks, each consisting of a WM phase with three trials, a distractor task, and an LTM phase. Within both the WM and LTM phases of each mini-block, participants were tested on three pairs, resulting in a total of 72 tests for each phase.

In the WM phase, each trial began with a fixation cross displayed at the center of the screen for 1,000 ms. During encoding, participants were shown three word pairs, each presented within one of three rectangles. Unlike Experiment 1, the word pairs were randomly assigned to locations rather than presented in a clockwise order. Each pair was displayed for 2,000 ms, separated by a 500-ms interval. In contrast to Experiment 1, we presented each pair only once to reduce potential reliance on LTM. The three empty rectangles remained visible throughout the subtrial.

The WM retrieval phase commenced immediately after presentation, as in Experiment 1. Participants heard two words separated by 2,000 ms and indicated whether they belonged to the same word pair (positive trial) or different pairs (lure trial). During retrieval, a digit stream of nine numbers appeared, with each digit shown for a variable duration between 700 ms and 3 s; the duration of each digit was drawn from a shifted exponential distribution. Participants pressed the space bar whenever the digit changed. The digit stream appeared in one of four locations: one of the three rectangles or the screen center. Its location could be congruent, incongruent, or central relative to the first word probe, as in Experiment 1. Participants received feedback on both the memory task and the digit-tracking task at the end of each trial.

After three WM trials, participants completed a 45-s mathematical distractor task. The LTM phase mirrored the WM retrieval procedure, testing word pairs presented in the three trials of the preceding WM phase. Each LTM test included one word pair from each WM trial, with trial order randomized. Recalibration of the eye tracker was conducted every eight mini-blocks.

### Data analyses and results

#### Eye movements

**Fig. 6:**
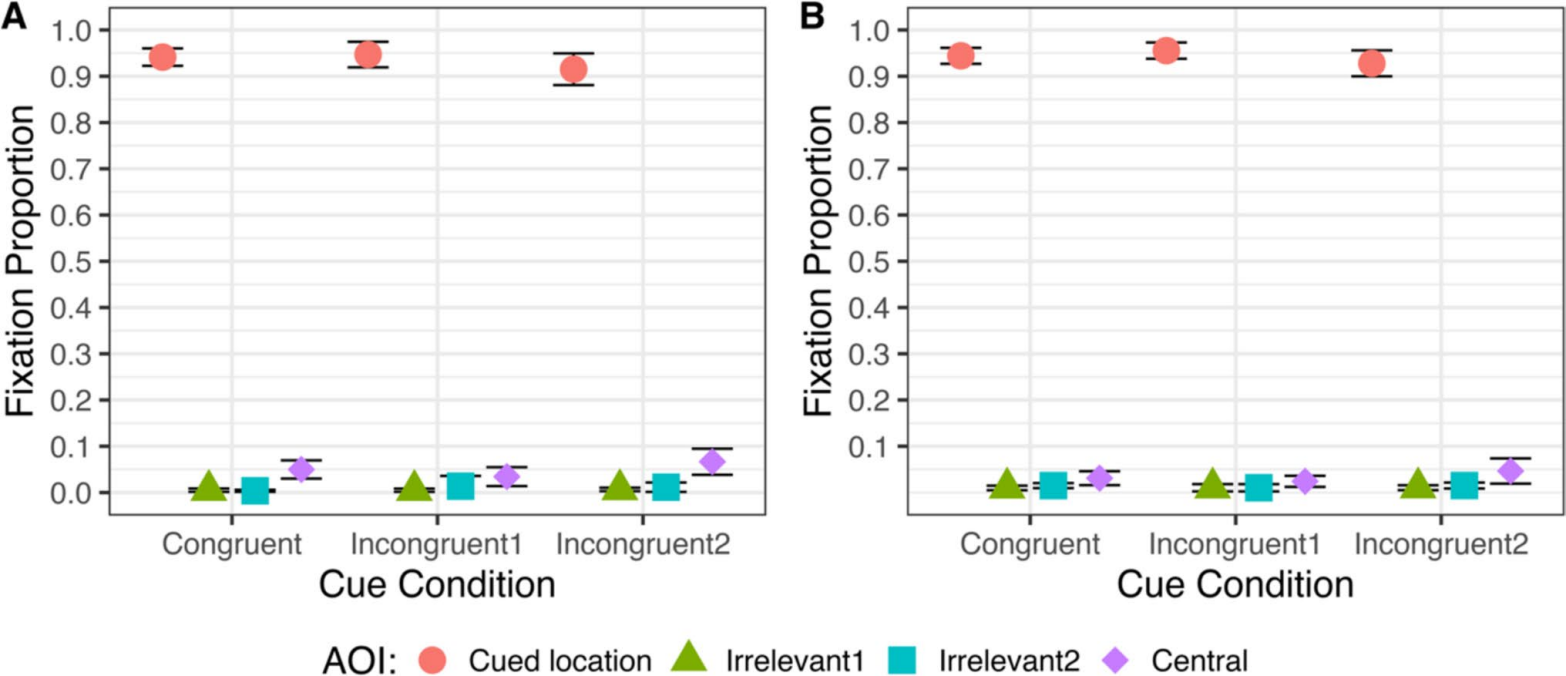
Fixation proportion to the four areas of interest during the retrieval phase. (**A**) working memory phase (WM) and (**B**) long-term memory (LTM) phase. The error bars indicate 95% within-subject confidence intervals. The error bars in Plot A are sufficiently small that they are obscured by the data points; nevertheless, they were computed and indicate very low variability in these conditions

The data analysis conducted for Experiment 2 is similar to that for Experiment 1. The size of the AOIs is 660 × 240 pixels, which is smaller than the AOIs in the first experiment, accounting for the smaller size of the rectangles. However, while estimating if participants followed the instructions, we only considered the AOIs relevant for the trial, that is, the three rectangles and the center of the screen. We first checked if participants followed the instructions of the task and looked at the locations where the cue was presented (Fig. 6). We conducted Bayesian ANOVAs for the same dependent variables as in Experiment 1. Participants followed the instructions and looked credibly more at the location with the cue than at locations without it in both the WM phase (BF_10_ = 4.0 × 10^34^) and the LTM phase (BF_10_ = 2.8 × 10^24^).

The mean accuracy for the digit-tracking task for the two blocks was satisfactory: 81% (*SD* = 0.7) for the WM phase and 72% (*SD* = 0.90) for LTM phase. Therefore, the performance in the digit-tracking task was a bit higher in the WM than in the LTM phase but overall similar to that in Experiment 1, indicating that the conditions were comparably difficult.

#### Memory performance

**Fig. 7:**
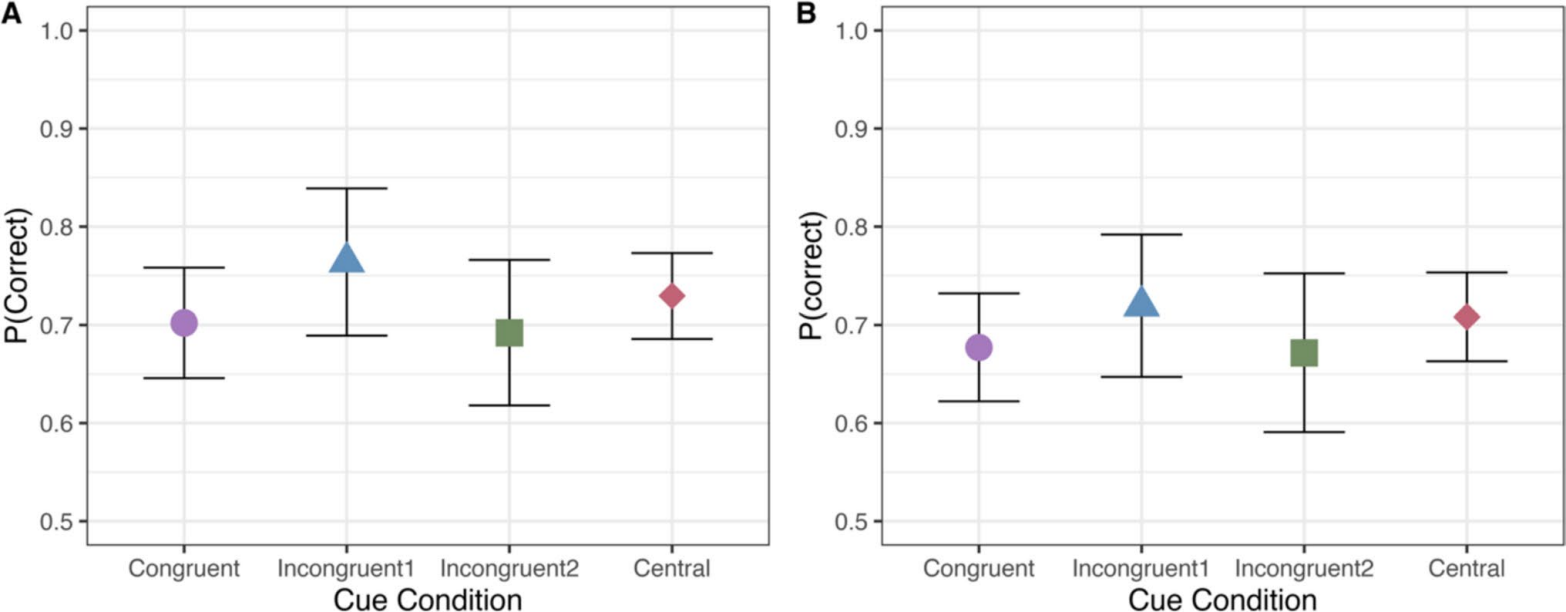
Proportion correct for the memory task across the cue conditions and phases. (**A**) Working memory (WM) phase and (**B**) long-term memory (LTM) phase. The error bars indicate the 95% within-subject confidence intervals

Figure 7 presents the proportion of correct responses across the two phases for the four cue conditions. Our results showed strong evidence against a main effect of cue for both WM (BF_10_ = 0.14) and LTM (BF_10_ = 0.16) phases.

Table 3 shows that we found evidence against a difference for most pairwise comparisons except for the comparison of central and incongruent conditions, for which we observed ambiguous evidence leaning against a difference.

**Table 3:** Bayes factor (BF_10_) for pairwise comparisons for the proportion correct across both the cue conditions and phases

Phase	Working memory		Long-term memory	
	Incongruent	Central	Incongruent	Central
Congruent	0.35	0.26	0.40	0.25
Incongruent		0.41		0.43

**Fig. 8:**
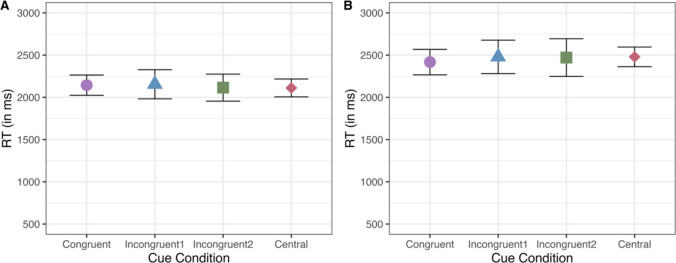
Reaction times for the memory task across the cue conditions and phases. (**A**) Working memory (WM) phase and (**B**) long-term memory (LTM) phase. The error bars indicate the 95% within-subject confidence intervals

To see if the benefit of the congruent condition and the cost of the incongruent condition was reflected in the time taken to respond to the task, we analyzed the RTs (Fig. 8). Our results speak against a main effect of cue for both WM (BF_10_ = 0.007) and LTM (BF_10_ = 0.005). The BFs for pairwise comparisons are shown in Table 4. We found evidence against a difference for all the pairwise comparisons. Thus, we did not observe a difference in memory performance between the different cue conditions.

**Table 4:** Bayes factor (BF_10_) for pairwise comparisons for the reaction times across both the cue conditions and phases

Phase	Working memory		Long-term memory	
	Incongruent	Central	Incongruent	Central
Congruent	0.09	0.08	0.07	0.10
Incongruent		0.07		0.06

**Fig. 9:**
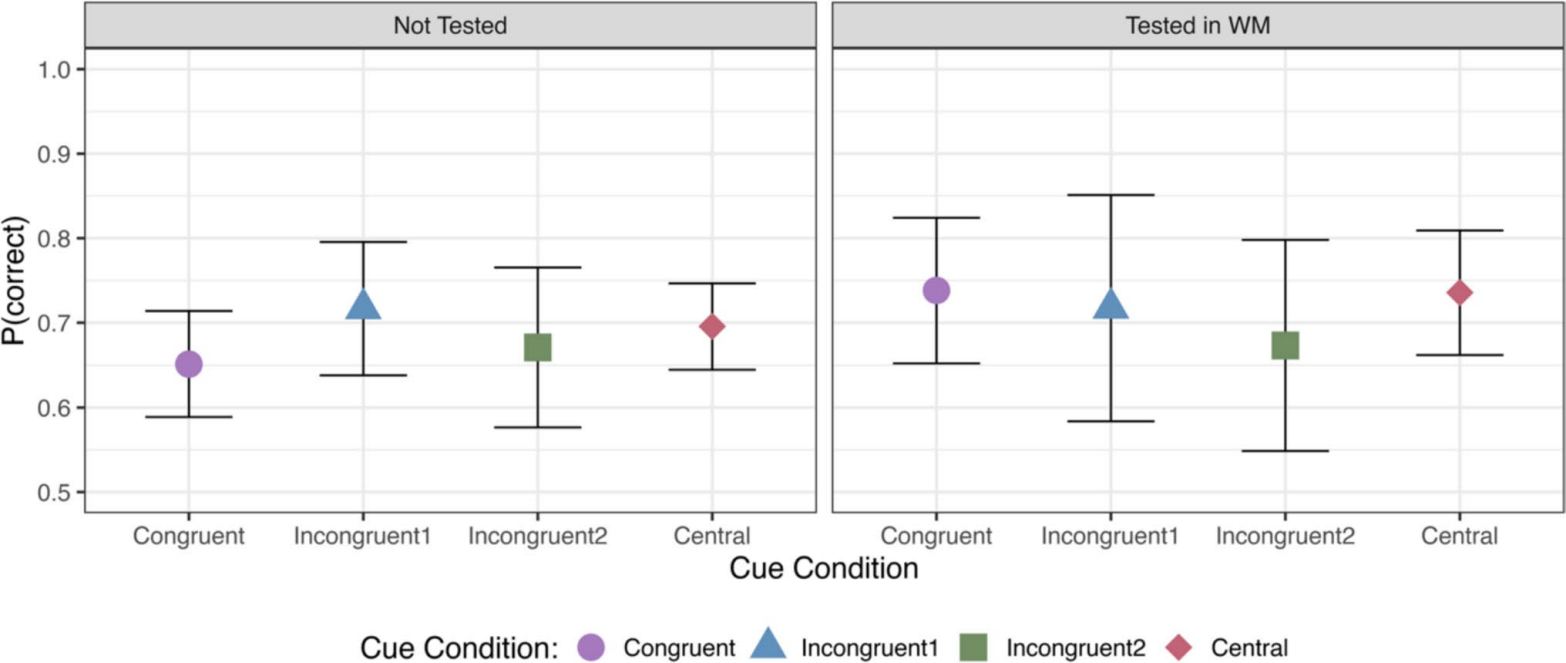
Proportion correct for long-term memory (LTM) across the cue conditions and tested items. The error bars indicate the 95% within-subject confidence intervals

As an exploratory analysis, we also looked if there was a difference in the effect of cues in the LTM phase in the subset of trials in which the word pairs were tested in WM and then retested in LTM. A tested word pair was defined as a pair for which one of its words had previously been presented as the first probe during the WM phase and was presented again during the LTM retrieval phase. The number of tested versus not tested pairs in each condition was not controlled for. Thus, the number of trials varies for the tested and the not tested pairs. For tested pairs we found a descriptively higher memory accuracy for the congruent and central condition as compared to the incongruent conditions but with no credible difference (Fig. 9, Table 5). The same was not observed for the not-tested pairs.

**Table 5:** Bayes factor (BF_10_) for pairwise comparisons of conditions for the accuracies in the long-term memory test separately for pairs tested and pairs not tested in the working memory phase

Test type	Tested		Non-tested	
	Incongruent	Central	Incongruent	Central
Congruent	0.38	0.19	0.46	0.30
Incongruent		0.34		0.36

## Discussion

In Experiment 2, we examined whether our proposition that LAN is functional for LTM retrieval but not for WM retrieval holds in an associative recognition task involving word pairs. Our results did not support this proposition: Memory performance did not improve when a cue was presented at a location congruent with the location associated with the to-be-retrieved pair. Alternative explanations are discussed in detail in the *General discussion*.

## General discussion

We examined whether the LAN effect is functional in WM and LTM by manipulating attention and eye movements toward encoding locations. In Experiment 1, evidence was ambiguous for accuracy but conclusive for RTs. In Experiment 2, however, we found strong evidence against the predicted cueing effects on both measures. Differences in retrieval timing between WM and LTM therefore cannot explain the absence of an effect, suggesting that LAN functionality depends on boundary conditions beyond memory system alone.

One potential factor is task difficulty. In Scholz et al. (2018), each sentence contained four attributes, creating substantial retrieval competition because participants had to decide whether a probe matched any attribute of the episode. In our task, each word was linked to only one other word, producing minimal competition. This may have enabled relatively rapid retrieval in both WM and LTM, leaving little opportunity for the indirect, location-based retrieval route mediated by LAN to contribute, which could explain the null effects.

A second factor may be the strength of item–location associations. Scholz et al. presented items for 9 s per location, whereas our pairs were shown for only 2 s. Longer exposure may foster stronger bindings, increasing the likelihood that location becomes a useful retrieval cue. Tentative support comes from a descriptive increase for congruent locations in Experiment 1, when the word pairs were presented twice at encoding, as well as a slight decrease in memory performance for incongruent locations when WM-tested items were retested in LTM in Experiment 2. The critical determinant may therefore be whether the association exceeds a threshold required to influence performance. As location was never part of the memory test and was only incidentally encoded, greater processing time may be necessary for LAN to become functional.

To conclude: Previous studies have shown that in some conditions that elicit LAN, attending to the empty location associated with a memorized event helps to retrieve it (Johansson & Johansson, 2014; Scholz et al., 2016, 2018). Our study shows that in at least one other condition that also elicits LAN (Bhanap et al., 2025b) this is not the case, demonstrating that sometimes LAN occurs without being functional. Although the former conditions involve tests of episodic LTM whereas the latter involves a test of WM, this distinction is not sufficient to predict whether LAN is functional or not.

## Data Availability

The data have been made available on the Open Science Framework at: https://osf.io/3gz8q/

## Footnotes

1 Our current study differs from other studies that investigate eye movements in WM during the delay period after a retro cue (Van Ede et al., 2019) and encoding (Czoschke et al., 2019). We investigated eye movements during retrieval, thereby addressing the potential causal role of eye movements to uninformative locations for retrieval.

2 In a pilot study, we presented participants with four word pairs, as in Bhanap and colleagues (2025a). The addition of the digit-tracking task during the retrieval phase led to chance-level memory performance. For that reason we reduced the set size to three pairs. The data and the analysis scripts for this pilot have been made available on OSF.

## Code availability

The R scripts for preprocessing and analyzing the data have been made available he Open Science Framework at: https://osf.io/3gz8q/
